# Zinc-Binding Cysteines: Diverse Functions and Structural Motifs

**DOI:** 10.3390/biom4020419

**Published:** 2014-04-17

**Authors:** Nicholas J. Pace, Eranthie Weerapana

**Affiliations:** Department of Chemistry, Boston College, 2609 Beacon Street, Chestnut Hill, MA 02467, USA; E-Mail: nicholas.pace@bc.edu

**Keywords:** zinc, cysteine, zinc-cysteine complexes, zinc fingers, zinc inhibition, regulatory zinc

## Abstract

Cysteine residues are known to perform essential functions within proteins, including binding to various metal ions. In particular, cysteine residues can display high affinity toward zinc ions (Zn^2+^), and these resulting Zn^2+^-cysteine complexes are critical mediators of protein structure, catalysis and regulation. Recent advances in both experimental and theoretical platforms have accelerated the identification and functional characterization of Zn^2+^-bound cysteines. Zn^2+^-cysteine complexes have been observed across diverse protein classes and are known to facilitate a variety of cellular processes. Here, we highlight the structural characteristics and diverse functional roles of Zn^2+^-cysteine complexes in proteins and describe structural, computational and chemical proteomic technologies that have enabled the global discovery of novel Zn^2+^-binding cysteines.

## 1. Introduction

**Figure 1:**
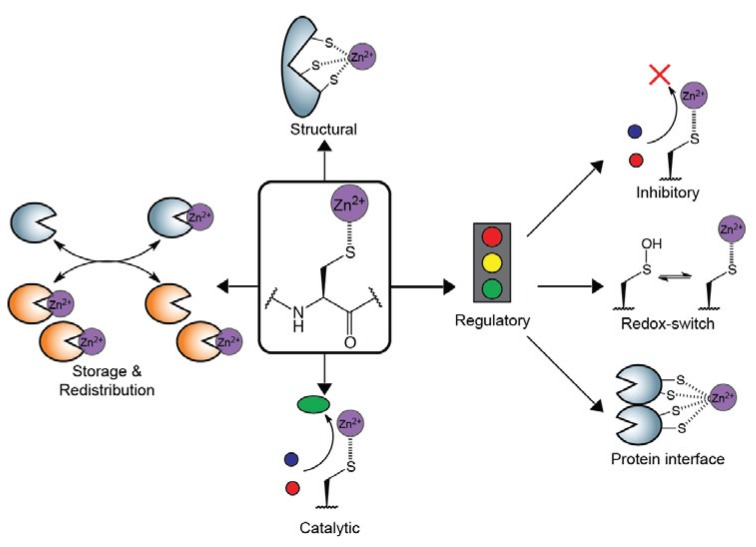
Zinc ions (Zn^2+^) have the ability to be chelated to cysteine residues within protein scaffolds. These resulting Zn^2+^-cysteine complexes participate in a variety of functional roles, including structural, catalytic, regulatory and transport. Regulatory mechanisms consist of inhibitory, redox-switches, and protein-interface stabilization.

When considering biologically relevant transition metals, zinc is the second most abundant found within cells, behind only iron. Zinc ions (Zn^2+^) are known to facilitate diverse protein functions that are essential for life. Common Zn^2+^ ligands found within proteins include cysteine (S), histidine (N), aspartate (O), and glutamate (O) residues. The ionization state of the thiol group of cysteine governs its ability to bind metals, including Zn^2+^. The pK_a_ of the thiol group of cysteine is typically close to physiological pH (7.4) [1]; therefore, the ionization state of cysteine is highly sensitive to small changes within the local protein environment [2]. Thus, the affinity of cysteine for Zn^2+^ varies accordingly for each individual cysteine within a protein scaffold. These resulting complexes contribute to protein structure, catalysis, and regulation (Figure 1) [3,4]. Zn^2+^-containing protein structural motifs are best highlighted by the well-characterized zinc fingers, first discovered over 25 years ago [5,6,7]. Although less common, Zn^2+^-cysteine complexes also catalyze enzymatic transformations in diverse classes such as oxidoreductases, transferases, and hydrolases. More recently, the potential for Zn^2+^ to modulate protein activities has been established. These regulatory complexes proceed through distinct mechanisms, such as Zn^2+^-inhibition, redox-switches, and stabilization of protein interfaces [8,9,10]. Lastly, the cysteine-rich metallothioneins tightly regulate cellular Zn^2+^ levels by storing and properly redistributing Zn^2+^ throughout the cell [11]. Due to these diverse functional roles of Zn^2+^-cysteine complexes, the development of both experimental and theoretical approaches has been paramount in the identification and characterization of Zn^2+^-binding cysteines. Here we summarize key examples of functional Zn^2+^-cysteine complexes, and discuss recent advances in computational and proteomic technologies to study these complexes.

## 2. Structural Zn^2+^-Cysteine Complexes: Zinc Fingers

**Figure 2:**
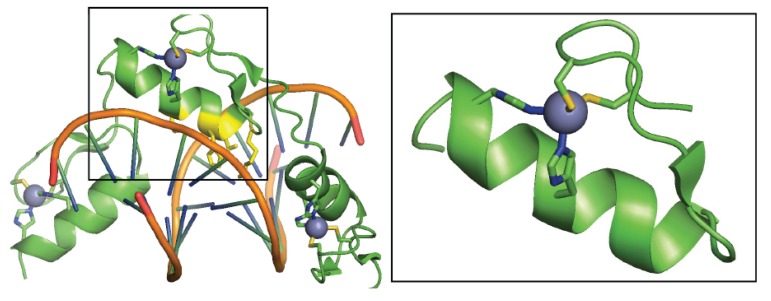
Three zinc fingers motifs bound within the major groove of a DNA strand with a single zinc finger motif being highlighted (black box). Zn^2+^ (purple) is bound to two cysteine and two histidine ligands within an α-helix and antiparallel β-sheet. Variable sequences at the −1, 2, 3, and 6 residues of the α-helix (yellow) dictate preferential binding to specific nucleotide sequences. (PDB ID: 1A1J).

Because zinc is a d10 transition metal, it exclusively forms a Zn^2+^ ion and typically lacks redox activity within cells. Zn^2+^ typically is found to assemble coordination complexes with four ligands in a tetrahedral geometry. Recent studies estimate the human proteome consists of approximately 3000 Zn^2+^-proteins [12]. Of the potential Zn^2+^ ligands within proteins, the sulfur atom of cysteine transfers the most charge over to the Zn^2+^. As cysteine occupies more ligand sites, it often quenches the ability of the Zn^2+^ to act as a lewis acid, rendering these complexes relatively inert [13]. As a result, Zn^2+^-cysteine complexes traditionally perform structural roles within proteins. The most abundant class of structural Zn^2+^-cysteine complexes is the zinc finger, and these have been extensively classified [14]. Zinc fingers are characteristically comprised of Cys_4_ or Cys_2_His_2_ coordination environments [12]. The classical Cys_2_His_2_ zinc finger chelates a single Zn^2+^ within an α-helix and antiparallel β-sheet (Figure 2 inset) [15]. Zinc finger domains are typically found in clusters of four or more within a single protein, and often structurally stabilize the protein for interaction with other proteins and biomolecules, such as DNA and RNA.

Although the functional roles of most zinc finger proteins are poorly understood, most annotated proteins act as transcription activators or suppressors [7]. A single zinc finger possesses four amino acids at the −1, 2, 3, and 6 positions of the α-helix that participate in hydrogen-bond interactions with 3–4 nucleic acids within the major groove of DNA (Figure 2) [16]. Different sequences at these four positions preferentially bind to distinct nucleic acid sequences with high affinity and selectivity [16]. Consequently, this motif has been exploited in the development of zinc finger endonucleases for genetic engineering. By conjugating specific arrays of zinc fingers to a promiscuous FokI endonuclease, DNA can be cut at an indicated sequence to disrupt, add, or correct the gene of interest [17]. The development of a conserved linker sequence was vital to the construction of polymeric zinc-finger endonucleases, requiring DNA sequences of up to 18 bp for recognition [18]. This advance provided enough specificity to target single genes within the human genome [19,20]. The expansion of synthetic zinc finger endonucleases has extended genetic engineering across diverse gene families [17].

## 3. Catalytic Zn^2+^-Cysteine Complexes

**Figure 3:**
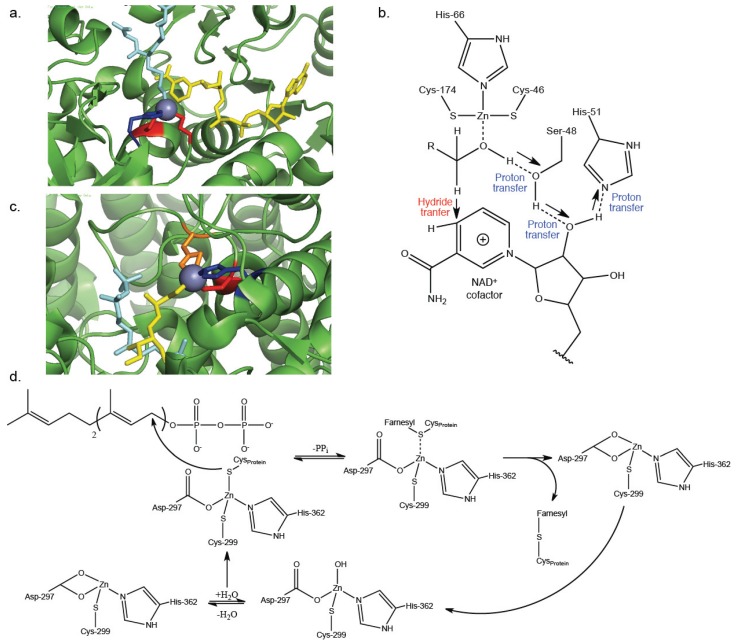
(**a**) The active site of ADH5 contains a Zn^2+^ (purple) bound to two cysteines (Cys46, Cys174, red) a histidine (His66, blue), and *S*-hydroxymethyl glutathione as the alcohol substrate (cyan). This positions the alcohol in the correct geometry to the adjacent NAD^+^ cofactor (yellow) (PDB ID: 1MC5); (**b**) The bound Zn^2+^ facilitates sequential proton transfers from the alcohol substrate to Ser48, NAD^+^, and His51, while also properly positioning the alcohol for a hydride transfer to NAD^+^. A generalizable “R” group is included in the structure of the primary alcohol, allowing for variability in the exact structure of the alcohol substrate. Figure adapted from Hammes-Schiffer and Benkovic [26]; (**c**) The active site of FTase contains a Zn^2+^ (purple) coordinated to Asp297 (orange), Cys299 (red), His362 (blue) and the thiol-containing target peptide (yellow) adjacent to the farnesyl diphosphate (cyan) (PDB ID: 1JCQ); (**d**) The cysteine of the target peptide is able to displace either an Asp297 or water ligand. The now-activated thiol forms a thioether linkage to the farnesyl group of farnesyl diphosphate, and is released by ligand exchange with Asp297 or water. Figure adapted from Ramos *et al.* [34].

Beyond structural roles, cysteines bind Zn^2+^ to directly facilitate enzymatic transformations. Cysteines are less common ligands in catalytic Zn^2+^ complexes due to the steric bulk of sulfur and greater charge transfer compared to histidine and water ligands [13]. However, catalytic Zn^2+^-cysteine complexes have been observed across diverse enzyme classes, such as oxidoreductases, hydrolases, and transferases (Table 1). The exact mechanism varies within each individual enzyme, but typically is comprised of either substrate coordination or activation by Zn^2+^. Alcohol dehydrogenase enzymes (ADH) were first discovered to require a Zn^2+^ for catalysis over 50 years ago [21]. These evolutionarily conserved enzymes facilitate the interconversion between alcohols and ketones or aldehydes. Humans possess six distinct classes of ADH enzymes (ADH1-ADH6), each utilizing the Zn^2+^-dependent catalytic mechanism [22]. The active enzyme is a dimer, with each 40 kD monomer possessing a substrate-binding Zn^2+^ and an NAD^+^ cofactor [23,24]. In the case of ADH5, the catalytic Zn^2+^ is bound to Cys46, His66, Cys174, and the alcohol substrate (Figure 3a) [25]. The bound Zn^2+^ coordinates the substrate in the correct geometry for the sequential proton transfer to Ser48 followed by hydride transfer to NAD^+^ (Figure 3b) [26]. Although they do not directly interact with the substrate, these cysteine residues are highly conserved throughout human ADH enzyme classes and are essential for ADH activity [22].

**Table 1:** Representative human proteins containing catalytic Zn^2+^-cysteine complexes.

Protein	Enzyme Class	Function	Mechanism	PDB Structure
Alcohol dehydrogenase	Oxidoreductase	Interconverts alcohols to aldehydes and ketones	Zn^2+^-coordination of substrate [23]	1MC5 [25]
Sorbitol dehydrogenase	Oxidoreductase	Reversible conversion of sorbitol to fructose	Zn^2+^-activation of nucleophilic water molecule [27]	1PL7 [27]
Cytidine deaminase	Hydrolase	Irreversible hydrolytic deamination of cytidine to uridine	Zn^2+^-activation of nucleophilic water molecule [28,29]	2KEM [30]
GTP cyclohydrolase I	Hydrolase	Converts GTP to dihydroneopterin triphosphate	Zn^2+^-activation of nucleophilic water molecule [31]	1FB1 [31]
Betain-homocysteine methyltransferase	Transferase	Transfers methyl group from betaine to homocysteine, forming dimethyl glycine and methionine	Zn^2+^-activation of thiol of homocysteine substrate [32]	1LT8 [32]
Protein farnesyltransferase	Transferase	Post-translational addition of farnesyl to cysteine residues within proteins	Zn^2+^-activation of thiol on target protein [33,34]	1JCQ [35]

While ADH acts through a Zn^2+^-substrate coordination mechanism, farnesyl transferase (FTase) relies on the activation of the substrate thiol by Zn^2+^ for its activity. FTase is part of the prenyltransferase protein family and catalyzes the post-translational addition of the 15-carbon farnesyl isoprenoid to proteins such as Ras, Rho, and Rab [36,37]. The isoprenoid is attached through a thioether linkage to a cysteine residue within a *C*-terminal CaaX peptide and is required for proper protein function by mediating membrane association and protein-protein interactions [38]. A Zn^2+^ is coordinated to Asp297, Cys299, and His362 within the active site of the β subunit of FTase (Figure 3c) [35]. The cysteine residue of the protein substrate coordinates to the Zn^2+^, displacing either a water or an Asp ligand. The adjacently bound farnesyl diphosphate is now vulnerable to nucleophilic attack by the Zn^2+^-activated thiol, resulting in the release of inorganic phosphate (PP_i_) and the farnesylated protein (Figure 3d) [33,34]. It’s important to note that ADH and FTase represent only two possible Zn^2+^-depended enzymatic mechanisms, and many others have been observed as well. In an alternative mechanism, cytodine deaminase employs a Zn^2+^ bound by a histidine, two cysteines, and a water molecule to irreversibly deaminate cytidine to uridine [30]. In this case, the water molecule becomes activated and acts as the nucleophile that facilitates the transformation [28,29]. These described catalytic mechanisms refute the common misconception that Zn^2+^-cysteine complexes are only capable of serving structural roles within proteins.

## 4. Regulatory Zn^2+^-Cysteine Complexes

Additionally, cysteine residues also have been observed to bind Zn^2+^ to modulate protein activities. In these cases, Zn^2+^-binding must be more transient in nature to allow for interchange between the bound and apo-forms. As a result, these cysteines are often more challenging to identify. Characterized regulatory mechanisms range in complexity, and have been categorized as inhibitory, redox-switches, and protein interface Zn^2+^-cysteine complexes (Table 2).

**Table 2:** Representative human proteins containing regulatory Zn^2+^-cysteine complexes.

Protein	Enzyme Class	Function	Mechanism	PDB Structure
Dimethylarginine dimethylaminohydrolase	Hydrolase	Converts N-omega,N-omega-methyl-L-arginine to dimethylamine and L-citrulline	Inhibitory [39]	2CI7 [40]
Ornithine transcarbamoylase	Transferase	Converts carbamoyl phosphate and ornithine to citrulline and phosphate	Inhibitory [41]	1EP9 [42]
Cathepsin S	Protease	Lysosomal cysteine protease	Inhibitory [8,43]	2HH5 [43]
Caspase 3	Protease	Cysteine protease	Inhibitory [44,45]	-
Caspase 6	Protease	Cysteine protease	Inhibitory [46]	4FXO [46]
Caspase 9	Protease	Cysteine protease	Inhibitory [47]	1JXQ [47]
Aconitase 2	Isomerase	Converts citrate to iso-citrate	Inhibitory [48]	-
Glutathione *S*-transferase omega	Transferase	Conjugates glutathione to a variety of electrophiles	Inhibitory [49]	-
Betain-homocysteine methyltransferase	Transferase	Transfers methyl group from betaine to homocysteine, forming dimethyl glycine and methionine	Redox-switch [32]	1LT7, 1LT8 [32]
Protein kinase C	Kinase	Phosphorylates serines and threonines	Redox-switch [50]	3PFQ [51]
Nitric oxide synthase	Oxidoreductase	Produces nitric oxide from arginine	Protein interface[52]; Redox-switch [53]	3NOS [52]
Apo2L/TRAIL	Cytokine	Induces signaling pathways to trigger apoptosis	Protein interface [54]	1DG6 [54]

### 4.1. Inhibitory Zn^2+^-Cysteine Complexes

Cysteine residues have been found to bind Zn^2+^ as a means of inhibiting enzymatic activity [8]. Inhibition usually occurs by chelation of Zn^2+^ to the catalytic cysteine residue, but allosteric inhibition attributed to Zn^2+^-binding at a cysteine distal to the active site has also been described (Table 2).

**Figure 4:**
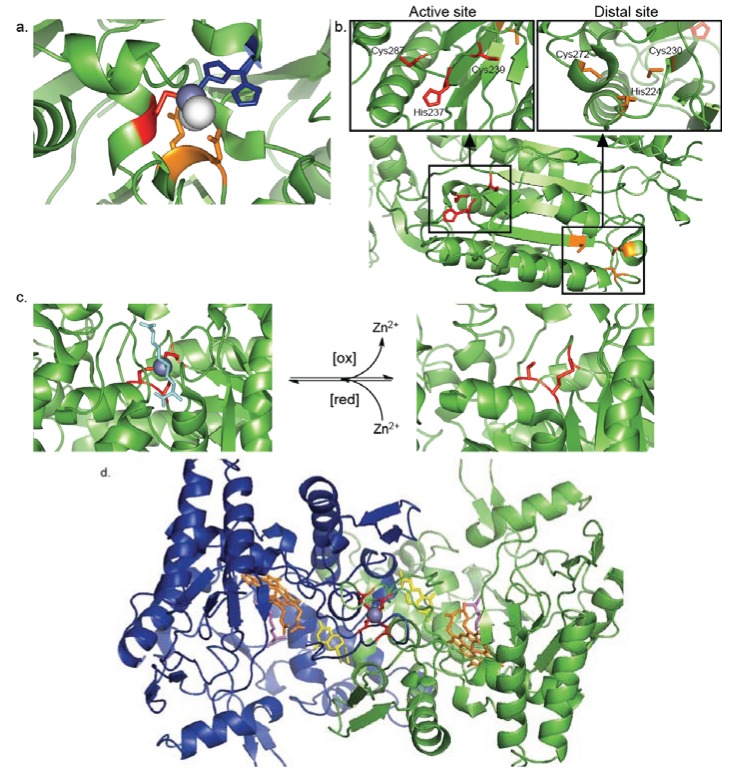
(**a**) The active site of dimethylarginine dimethylaminohydrolase (DDAH-1) is capable of binding Zn^2+^ to inhibit activity. Zn^2+^ (purple) is bound to the catalytic Cys274 (red) and His173 (blue). The remaining two ligands consist of water molecules (white) stabilized by hydrogen-bonding to Asp79 and Glu78 (orange) (PDB ID: 2CI7); (**b**) Caspase-9 has been shown to bind Zn^2+^ at the catalytic dyad consisting of Cys239 and His237, along with Cys287 (red), and also to Cys272, Cys230, and His224, (orange), which is found distal to the active site, to inhibit enzyme activity (PDB ID: 1JXQ); (**c**) Under reducing conditions, betain-homocysteine methyltransferase (BHMT) binds a Zn^2+^ (purple) to Cys217,Cys299, and Cys300 (red). The Zn^2+^-cysteine complex coordinates the substrate homocysteine (cyan) to initiate catalysis (left, PDB ID: 1LT8). When exposed to oxidizing conditions, Cys217 and Cys299 (red) form a disulfide bond, resulting in the release of Zn^2+^ from the active site (right, PDB ID: 1LT7); (**d**) The α-subunit (green) and β-subunit (blue) of endothelial nitric oxide synthase (NOS3) are dependent on a Zn^2+^-binding cysteines for dimerization. Cys94 and Cys99 (red) from each subunit chelate a Zn^2+^ (purple) to stabilize dimer formation, allowing for proper binding of the heme cofactor (orange), tetrahydrobiopferin cofactor (yellow), and homo-arginine substrate (magenta) within each active site (PDB ID: 3NOS).

Dimethylarginine dimethylaminohydrolase (DDAH-1) is a metabolic enzyme responsible for the conversion of dimethylarginine to dimethylamine and citrulline. Dimethylarginine is known to inhibit nitric oxide synthases to mitigate the production of nitric oxide, an important cell signaling molecule [55]. The most well-studied DDAH-1 is from bovine, however, the human homologue retains 94% sequence homology. Zn^2+^ inhibits DDAH-1 activity with a K_i_ of 4.2 nM at pH 7.4 [39]. This value is rather high when considering the physiological range of available Zn^2+^ concentrations and is suggestive of a weaker, more transient binding mode that is indicative of a regulatory role for Zn^2+^ within DDAH-1. The enzyme functions through a nucleophilic cysteine residue (Cys274) conserved in both the human and bovine proteins [56]. Structural studies reveal a Zn^2+^ bound to the catalytic Cys274 and His173 within the active site of the enzyme (Figure 4a) [40]. The remaining two ligands are comprised of water molecules stabilized by hydrogen-bonding to adjacent Asp79 and Glu78. DDAH-1 only possesses two Zn^2+^ ligands instead of the typical three or four, which may contribute to the weaker, more transient Zn^2+^ binding.

Although most inhibitory Zn^2+^-cysteine complexes are found to bind directly to the nucleophilic cysteine residue, the potential for allosteric inhibition has been realized in the case of Caspase-9. Caspases are cysteine-dependent aspartate-directed proteases that play a prevalent role in signaling cascades culminating in apoptosis [57]. Zn^2+^ has been implicated as a strict mediator of apoptosis, where small fluctuations in concentration can strongly dictate cell survival or death [58]. Caspase-9 is an initiator caspase that goes on to cleave caspase-3 and 7 to trigger apoptosis. When attempting to decipher the mechanism of Zn^2+^-mediated inhibition of Caspase-9, two distinct Zn^2+^ binding sites were uncovered. The first consisted of the catalytic dyad, His237 and Cys239, along with the adjacent Cys287, and was primarily responsible for the Zn^2+^-mediated inhibition [47]. The second binding site, which comprised Cys272, Cys230 and His224, was found distal to the active site (Figure 4b). Subsequent assays suggested that this distal site may have the potential for Zn^2+^-mediated allosteric inhibition of Caspase-9 activity [47]. To give precedence to this notion, Zn^2+^-mediated allosteric inhibition has been observed in Caspase-6, however cysteines are not involved in Zn^2+^ coordination in this instance [46].

### 4.2. Redox-Switch Zn^2+^-Cysteine Complexes

Cysteine residues are susceptible to a myriad of post-translational modifications including oxidation, nitrosylation, and disulfide formation [59,60,61]. Cysteine’s ability to bind Zn^2+^ is predicated upon the presence of a fully reduced, unmodified thiol. Thus, cellular redox metabolism can often be coupled to Zn^2+^-binding, giving rise to a “redox-switch” regulatory mechanism: increases in oxidants of sulfur release Zn^2+^, while reductants restore the Zn^2+^-binding capacity of the thiol [9]. Redox-switch Zn^2+^-cysteine complexes have been found to modulate diverse enzyme activities (Table 2). Betain-homocysteine methyltransferase (BHMT) is an essential metabolic enzyme that contributes to the biosynthesis of glycine, serine, threonine, and methionine [62]. This transformation relies on a Zn^2+^-cysteine complex to activate the homocysteine substrate. Under reducing conditions, Cys217, Cys299, and Cys300 chelate Zn^2+^ to give the active form of the enzyme (Figure 4c, left) [32]. Upon exposure to oxidative conditions, Cys217 and Cys299 form a disulfide bond resulting in the release of Zn^2+^ and inactivation of the enzyme (Figure 4c, right) [32]. This interplay between Zn^2+^-binding and disulfide formation couples the intracellular redox state to BHMT activity.

### 4.3. Protein Interface Zn^2+^-Cysteine Complexes

Zn^2+^-cysteine complexes can also bridge two proteins or protein-subunits. The dependence of protein-protein interactions on available Zn^2+^ levels establishes a novel mechanism to modulate protein supramolecular assembly and subsequent enzymatic activities (Table 2). Nitric oxide synthases (NOS) catalyze the formation of nitric oxide and citrulline from arginine through a complex mechanism consisting of five single-electron transfers [63]. Proper dimer formation is essential for oxidoreductase activity. Structures of the endothelial NOS isoform (NOS3) revealed a Zn^2+^ bound to Cys94 and Cys99 from each monomer (Figure 4d) [52]. The Zn^2+^-cysteine complex catalyzes proper dimer formation, a prerequisite for proper binding of the substrates and cofactors. Additionally, these Zn^2+^-binding cysteines appeared susceptible to redox-modifications, particularly by peroxynitrite. A recent study speculates that peroxynitrite facilitates disulfide-bond formation between Cys94 and Cys99 in each monomer, allowing for subsequent release of Zn^2+^, formation of free monomers, and disruption of enzyme activity [53]. This Zn^2+^-cysteine complex, employing both protein interface and redox-switch mechanisms, illustrates the potential for multifaceted protein regulation by Zn^2+^-binding cysteines.

## 5. Zn^2+^-Cysteine Complexes for Zn^2+^ Transfer & Cellular Redistribution

Because Zn^2+^ readily forms stable coordination complexes, free Zn^2+^ concentrations are found to be extremely low [64,65]. On the contrary, total cellular Zn^2+^ concentrations have been estimated on the order of 100 micromolar with Zn^2+^ being strongly buffered through a protein storage system [66]. Metallothioneins are a superfamily of low molecular weight proteins (6–7 kD) that possess 20 cysteine residues capable of binding up to 7 Zn^2+^ in the form of Zn_4_Cys_11_ and Zn_3_Cys_9_ clusters with unique geometries. These clusters have been evaluated as thermodynamically stabile, yet kinetically labile [67]. As a result, metallothionein and the apo-form, thionein, are able to rapidly donate/accept Zn^2+^ through ligand exchange [68]. This rapid exchange allows metallothioneins to increase the pool of available Zn^2+^ and provide an adequate source of Zn^2+^ for proteins [69]. Interestingly, while Zn^2+^-binding to metallothioneins has not been found to be cooperative, the cysteines of the Zn_4_S_11_ bind slightly tighter than the Zn_3_S_9_ cluster, producing a more fluid buffering mechanism [65]. Zn^2+^-complexes regulated by metallothioneins/thioneins modulate diverse protein activities such as gene expression and DNA repair [70].

## 6. Methods of Identification of Zn^2+^-Cysteine Complexes

**Figure 5:**
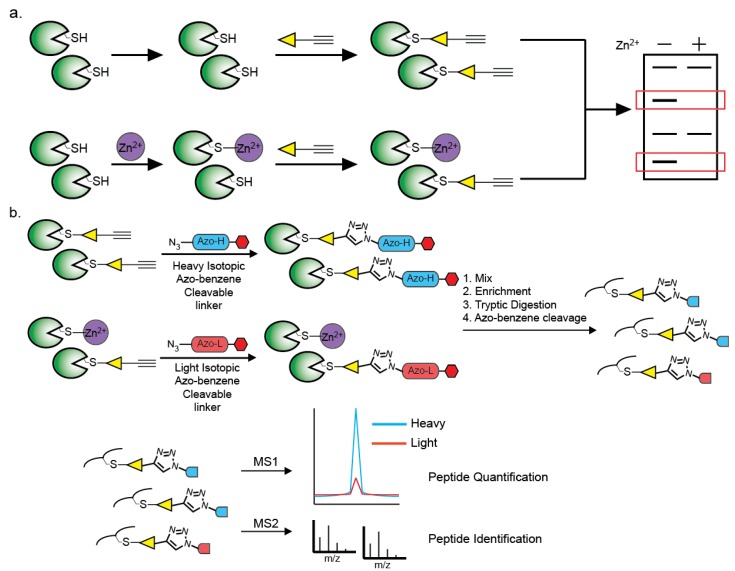
(**a**) A chemical-proteomic platform to identify Zn^2+^-binding cysteines. Zn^2+^ is added into one proteome, while another population acts as a control. Both are subsequently treated with a cysteine-reactive chemical probe. These probes possess an electrophilic warhead that preferentially binds to the more nucleophilic, apo-form of the protein. Through the use of the alkyne handle, the platform can be coupled to gel or mass spectrometry-based analytical techniques to identify the affinity of cysteines for Zn^2+^; (**b**) The platform can be adapted to quantitative mass spectrometry through the use of isotopic, chemically cleavable azobenzene biotin tags (Azo-H and Azo-L). The Azo-H is conjugated to the control population, while the Azo-L is added to the Zn^2+^-treated population. The two populations are then mixed, enriched on streptavidin beads, and digested by trypsin. After washing, the probe-modified peptides are chemically cleaved from the beads using sodium dithionite and analyzed by LC/LC-MS/MS. Light/heavy ratios are generated for each peptide and provide a quantitative measure of Zn^2+^ affinity for each modified cysteine.

Because Zn^2+^-binding cysteines play such essential physiological roles, strategies to identify and functionally characterize them have been thoroughly explored. The most common methods combine experimental approaches, such as structural genomics and protein NMR, and theoretical approaches, including homology searches of sequence databases [71,72,73]. These methods prove to be well-suited to distinguish Zn^2+^-binding cysteines within motifs where the structural features have been well-defined, such as zinc finger domains. However, regulatory Zn^2+^-cysteine complexes are more difficult to identify due to their necessary transient binding. By nature, these complexes must be more labile to allow for interchange between the Zn^2+^-bound and apo-protein forms. The employment of fewer protein-based ligands (one or two instead of three or four) and the use of ligands from multiple proteins or subunits at binding interfaces contribute to this transient binding ability. As a result, regulatory Zn^2+^-cysteine complexes are difficult to anticipate, and structures and homology searches fail to sufficiently detect them. Structure-based methods also require a high-resolution crystal structure of the protein of interest or a close homologue, and are therefore currently unable to access the entire proteome. With the ever-increasing number of recognized Zn^2+^-chelating proteins the ability to globally evaluate Zn^2+^-binding within a complex proteome has become paramount. Toward this end, a recent study developed a chemical-proteomic platform that serves as a valuable complement to previous approaches (Figure 5a) [49]. This platform exploits the reduced nucleophilicity of cysteine residues upon metal-binding by utilizing cysteine-reactive chemical probes that preferentially bind the more nucleophilic apo-form. Coupling these cysteine-reactive probes to gel and mass spectrometry-based proteomic techniques facilitates identification and quantification of the affinity of each cysteine towards Zn^2+^. A peptide-based probe was able to identify the Cys44 of sorbitol dehydrogenase (SORD), a known ligand of the catalytic Zn^2+^-cysteine complex, as means of validating the approach. Alternatively, the catalytic Cys32 of glutathione *S*-transferase omega 1 (GSTO1) was identified as a potential regulatory Zn^2+^-binding cysteine functioning through an inhibitory mechanism. The platform was extended by applying a promiscuous cysteine-reactive probe to globally identify putative Zn^2+^-binding cysteines across ~900 cysteines in the human proteome (Figure 5b). This strategy employs isotopic, chemically cleavable azobenzene biotin tags (Azo-H & Azo-L) [74] that are conjugated to the Zn^2+^-treated and control proteomes. The populations are enriched on streptavidin, mixed, and digested with trypsin. The remaining probe-modified peptides are cleaved from the beads and analyzed by LC/LC-MS/MS. Light/heavy ratios are generated for each peptide and provide a quantitative measure of Zn^2+^ affinity for each modified cysteine. This proteomic study identified several well-characterized Zn^2+^-binding proteins, such as ADH5, as well as numerous uncharacterized proteins from functionally distinct classes. For example, Cys385 of Aconitase 2 (ACO2) was identified as Zn^2+^ binding. While ACO2 has demonstrated mitigated activity upon Zn^2+^ treatment, the mechanism of inhibition is unknown [48]. This platform suggests Zn^2+^ binds to a cysteine and disrupts the assembly of an iron-sulfur cluster essential for the enzyme’s activity. Notably, this platform is more adept to identify regulatory Zn^2+^-binding cysteines because it is dependent on the presence of a certain population of the apo-protein. On the contrary, cysteines with stronger Zn^2+^ binding, such as Zn^2+^ fingers, may be more difficult to detect. This platform appears well-suited to complement previous methods to globally characterize the Zn^2+^-cysteine complexes.

## 7. Perspective and Conclusions

While traditionally Zn^2+^-cysteine complexes were thought to contribute solely to protein structure through zinc finger motifs, it is now apparent that these complexes are essential for protein catalysis and regulation. Catalytic transformations that utilize Zn^2+^ proceed through a variety of different mechanisms, allowing for the potential of discovering alternate mechanisms not currently known. Zn^2+^-cysteine complexes also regulate protein activities through sophisticated mechanisms, including inhibition, redox-switching, and protein interface stabilization and new modes of Zn^2+^-based regulation are constantly being unveiled. The advent of structural, computational, and proteomics methods have accelerated these discoveries. Further developments in these technological platforms will help uncover more intricate and multifaceted catalytic and regulatory processes that are currently unannotated. The development of new methods is vital as we aim to expand the scope of Zn^2+^-cysteine complexes and their functional roles within proteins across the entire proteome.
